# GIT1 overexpression promotes epithelial-mesenchymal transition and predicts poor prognosis in hepatocellular carcinoma

**DOI:** 10.1080/21655979.2020.1855914

**Published:** 2020-12-21

**Authors:** Guifu Wang, Xuesong Bai, Guoqing Jiang, Shengjie Jin, Qian Wang, Aoqing Wang, Rui Peng, Aiwu Ke, Dousheng Bai

**Affiliations:** a Department of Hepatobiliary Surgery, Clinical Medical College, Yangzhou University, Yangzhou, Jiangsu, P.R. China; b The First Clinical Medical College, Dalian Medical University, Dalian, Liaoning, P.R. China; c Liver Cancer Institute, Zhongshan Hospital, Fudan University, Shanghai, P.R. China

**Keywords:** Hepatocellular carcinoma, GIT1, EMT, ERK1/2 signaling, prognosis

## Abstract

Globally, hepatocellular carcinoma (HCC) is one of the most common causes of cancer-associated mortalities. It has a high rate of metastasis and recurrence, which predict a poor prognosis. G-protein-coupled receptor (GPCR)-kinase interacting protein-1 (GIT1) is a multifunctional scaffold protein that mediates the progression of various tumors. Studies have correlated GIT1 with HCC, however, these correlations have not been fully elucidated. Therefore, we aimed at evaluating the expression of GIT1 in HCC tissues and cells, and to investigate its role and potential mechanisms in HCC progression. The expression levels of GIT1 in HCC tissues and other cancers was determined by using the Oncomine and TCGA databases. Functional analysis of GIT1 in HCC was evaluated through *in vitro* and *in vivo* experiments, whereby, HCC cells were transfected with synthetically overexpressed and short hairpin RNA (shRNA) lentivirus-mediated plasmids. Kaplan–Meier and Cox regression methods were used to establish the associations between GIT1 and clinical outcomes of 158 HCC patients. GIT1 was found to be elevated in HCC tissues where it promoted the invasion, migration, and proliferation of HCC cells. Moreover, the overexpression of GIT1 prompted epithelial-mesenchymal transition (EMT) by activating extracellular regulated kinase 1/2 (ERK1/2) pathway, which was shown to be reversed by SCH772984, a specific ERK1/2 inhibitor. GIT1 was also found to be associated with malignant features of HCC, leading to a poorer prognosis. In conclusion, GIT1 promotes HCC progression by inducing EMT and may reflect the course of HCC patients.

## Graphical abstract

**Figure:**
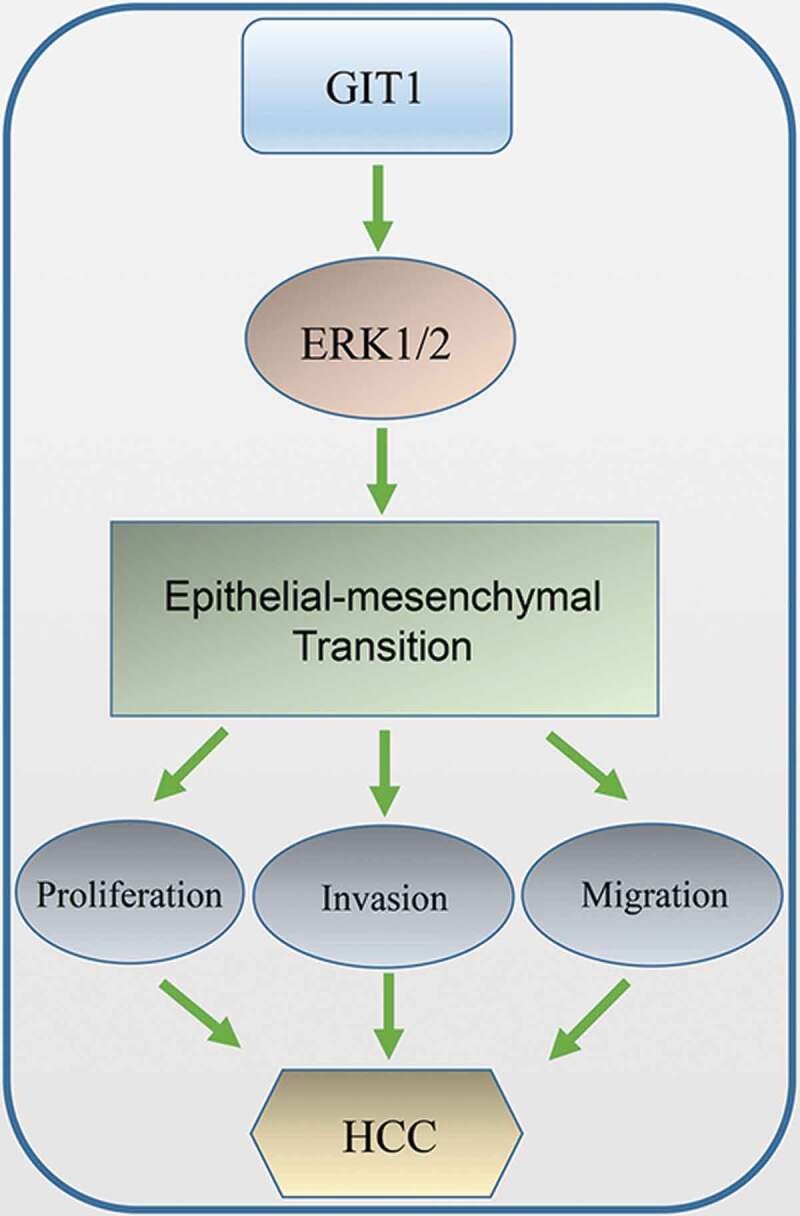

## Introduction

Globally, hepatocellular carcinoma (HCC) is the 6^th^ most common cancer and the 4^th^ most common cause of cancer-associated mortalities [1,2]. Advances in cancer screening, diagnosis and management have improved HCC prognosis [3]. However, high metastatic and recurrence incidences hamper HCC survival rates after surgical resection [4,5]. Therefore, elucidating the pathogenesis and mechanisms of HCC will provide potential avenues for HCC clinical management.

As a multifunctional scaffold protein, GIT1 (G-protein-coupled receptor kinase interacting protein-1) was initially considered to be an ADP ribosylation factor GTPase-activating protein (ARFGAP) that suppressed beta2-adrenergic receptor signaling and promoted receptor phosphorylation [6,7]. Several kinds of molecules have been confirmed to interact with GIT1 through its multiple domains [6]. Studies have documented that GIT1 functions in nerve, blood vessel, and bone. Overexpression of GIT1 promotes neurite outgrowth and spine maturation in nervous tissues [8–10]. It is also associated with attention deficit hyperactivity disorder (ADHD) [11]. In blood vessels, GIT1 is involved in the regulation of vascular intima formation and is required for pulmonary vascular development [12,13] While in bone metabolism, GIT1 silencing suppresses chondrocyte proliferation and apoptosis, delaying fracture healing [14].

Notably, GIT1 is critical for focal cell migration, adhesion, and lamellipodia formation [15–17]. It has also been found to modulate multiple pathways, including MEK1/2-ERK1/2 [18], NF-κB and Notch signaling pathways [19]. Moreover, GIT1 is elevated by integrin-β1 to regulate chondrocyte proliferation and apoptosis [20], but in osteoclast, GIT1 functions in promoting autophagy by disrupting Beclin-1-Bcl-2 binding during starvation [21]. These GIT1 properties enhance its involvement in cancer occurrence and development. GIT1 has been reported to be upregulated in several malignant tumors, such as oral, lung, breast, and gastric cancer [22–25]. However, its roles and mechanisms in HCC need further elucidation.

Therefore, this study aimed at evaluating the expression levels of GIT1 in HCC tissues, and to investigate the effects and definitive mechanisms of GIT1 in promoting HCC progression. We also confirmed the relationship between GIT1 and the prognosis of HCC patients.

## Methods and materials

### Patients and samples

A total of 28 pairs of frozen tissues and matched normal tissues were obtained from Zhongshan Hospital (Shanghai, China) and analyzed by quantitative reverse transcription-polymerase chain reaction (qRT-PCR). 8 sample pairs were chosen for western blot analysis. A total of 158 HCC patients undergoing complete surgical resection at Zhongshan Hospital Fudan University (Shanghai, China) between January 2006 and December 2008 were randomly recruited into this study. HCC diagnosis was confirmed by 2 independent pathologists. Tumor and paracancerous tissues were acquired after surgery and analyzed by tissue microarray. All participants gave a written informed consent. Ethical approval for this study was obtained from the institutional review board of Zhongshan Hospital Fudan University.

### Tissue microarray

First, H&E staining was used to histologically identify HCC samples before representative non-necrotic and non-hemorrhagic areas were selected. In each case, 1-mm-diameter repeated punches were obtained from 2 different areas, the tumor center and the adjacent non-cancerous edge (respectively designated as intratumor and adjacent to cancer; 4 holes in total), to ensure reproducibility and uniform staining (Shanghai Biochip Co., Ltd., Shanghai, China). Thus, four tissue microarray blocks were prepared, each comprising 158 cylinders. The 4 µm thick cross-section was then mounted on a glass slide coated with 3-aminopropyltriethoxysilane.

### Immunohistochemistry

Formalin-fixed and parrffin-embedded (FFPE) HCC and adjacent normal tissue sections, as well as the TMAs were analyzed by immunohistochemistry (IHC). Sections were incubated at 60°C for 2 h and dewaxed in xylene. Rehydration was done in descending alcohol concentrations. Inactivation of endogenous peroxidases in the sections was performed by treating then with 0.3% hydrogen peroxide for 15 minutes at room temperature. Sections were then microwaved in EDTA for 15 minutes for antigen retrieval. They were then blocked with 1% BSA for 30 minutes followed by an overnight incubation with rabbit anti-GIT1 at 4°C (Abcam, Cat. No. ab171956) at 1:100. Next, sections were washed in PBS and incubated with HRP-conjugated secondary antibody (Goldenbridge Biotechnology, Cat. No. PV-9000) at room temperature for 20 mins. Samples were counterstained with hematoxylin and signal developed with diaminobenzidine. They were then dehydrated in alcohol and cleaned using xylene before mounting.

### Evaluation of immunostaining

Tissues were stained and imaged. Staining intensity was scored as: 3 = strongly positive, 2 = moderately positive, 1 = weak positive, 0 = negative while positive staining percentage was scored as: 4 ≥ 75%, 3 = 50–75%, 2 = 25–50%, and 1 ≤ 25%. The total score ranged between 0–2 for negative staining and 3–12 for positive staining. All sections were scored by 2 independent pathologists.

### Western blot analysis

Tissue and cellular proteins were extracted and quantified. A 10% polyacrylamide gel was used to resolve the proteins. Proteins were transferred onto 0.2 µm PVDF membranes (Merck Millipore, Germany). GAPDH levels were used to normalize loading. A solution of 10% fat-free milk in TBST was used to block the membrane for 2 h at room temperature after which the membranes were rinsed. The proteins were probed using antibodies at 4°C overnight. These antibodies included rabbit anti-human GIT1 (Abcam, Cat. No. ab171956); rabbit anti-p-ERK (Cat. No. #4370), anti-ERK (Cat. No. #4695), anti-E-cadherin (Cat. No. #3195), anti-p-AKT (Cat. No. #4685), anti-Vimentin (Cat. No. #5741), anti-β-cadherin (Cat. No. #8480), anti-AKT (Cat. No. #9272), and anti-GAPDH (Cat. No. #5174), all bought from Cell signaling, and diluted to 1:1000. Membranes were then incubated with HRP-conjugated secondary antibody (Cell signaling, Cat. No. #7074) at 1:2000 for 2 h at room temperature. Chemiluminescence was developed using enhanced ECL chemiluminescent substrate (New Cell & Molecular Biotech, Suzhou, China) and membranes imaged on Chemidoc XRS Gel Imaging System (Biorad). Images were analyzed using ImageJ software.

### Quantitative reverse transcription polymerase chain reaction

Total RNA was extracted from frozen tissue samples using the TRIzol reagent (Invitrogen, USA). cDNA was synthesized from the extracted total RNA using prime Script RT reagent kit (Takara, Japan). SYBR Premix Ex Taq (Yeasen, Shanghai, China) was used for qPCR. Experiments were repeated 3 times. The following primers were used: GIT1 primers: fwd: 5′-CAGCCTTGACTTATCCGAATTG-3′, rvs: 5′-ACACTGCATCATCTTCTCTTCG-3′, GAPDH: fwd: 5′-ATGACCCCTTCATTGACCTCA-3′, rvs 5′-GAGATGATCACCCTT TTGGCT-3′. GAPDH was used as the reference gene.

### Cell lines and cell culture

HCC cell lines (Huh7 and MHCC97-H) were acquired from the Chinese Academy of Sciences Shanghai branch cell bank (Shanghai, China). They were cultured on DMEM (Gibco, USA) supplemented with 10% FBS (Gibco, USA) and pen/strep. Cell cultures were incubated in a humified incubator at 37°C in 5% CO_2_. Where indicated, cells were cultured in medium containing 20 μM SCH772984 (MedChem Express, USA) for one day before cell harvesting and protein extraction.

### Lentivirus-mediated plasmid transfection

Lenti-GIT1 cDNA vector, lenti-shGIT1 vector, and corresponding control vectors were generated by Obio Technology Company (Shanghai, China). Huh7 cells were cultured in 6-well plates to ≤50% confluence. They were then transfected with the lenti-GIT1 cDNA vector and its control vector. MHCC97-H cells were transfected with a lenti-shGIT1 vector and its control vector. GIT1 shRNA target sequences were: shRNA1: 5ʹ-GCTGGTTGAGTGCCAATAT-3ʹ; shRNA2: 5ʹ-CCACCTTGATCATCGACAT-3ʹ; shRNA3: 5ʹ-GCACAGAGGATGTCATCTT-3ʹ. Cells were cultured in medium containing 5 μg/ml polybrene (Obio Technology) for 24 h. Stable clones were selected using puromycin (Beyotime Institute of Biotechnology, Shanghai, China) for 3 days. GIT1 expression was assessed by western blot.

### Cell counting Kit 8 assay

The cell counting kit 8 (CCK-8) (Yeasen, Shanghai, China) was used to determine cell proliferation. Briefly, 2,000 cells in 100 µL were seeded into each well of a 96-well plate. Cell viability was evaluated every 24 h for 4 days by adding 10 μl of CCK-8 substrate into each well and incubating in normal culture conditions for 2 h before absorbance was read at 450 nm using an automatic plate reader.

### Clone formation

Briefly, 1,000 cells were seeded in each well of a 6-well plate and cultured for 2 weeks under normal conditions. They were then washed thrice using PBS 1X and fixed in 4% paraformaldehyde (PFA), before staining with 0.1% crystal violet. The number of colonies was microscopically counted.

### Wound healing assay

A total of 2 × 10^5^ transfected cells were seeded into each well of a 6-well plate. At 100% confluence, a scratch wound was created using a 200 μL tip. They were then incubated in serum-free medium. Cell migration distance was calculated by measuring wound width shortening at 0 and 48 h. Cells were imaged under a microscope at appropriate times. Image J was used to calculate the average cell migration area. Three independent assays were performed.

### Transwell invasion assays

Eight μm Transwell chambers (Corning, USA) with a matrix gel were used in the transwell invasion assays. Cell density of 5 × 10^4^ cells/well in serum-free medium were inoculated in the upper chamber. A complete media (containing 10% FBS) was inoculated in the lower chamber and incubated for 48 h. Thereafter, cells obtained from the upper chamber were washed. Those that migrated to the lower chamber were fixed in methanol, stained using 0.1% crystal violet, and counted. Three independent assays were performed.

### Tumorigenesis in vivo

Twelve 4-week-old nude mice housed in standard conditions were alloctaed into 4 groups. 5 × 10^6^ HCC cells were subcutaneously injected into the flank of mice. Tumor volume was monitored weekly and after 4 weeks, the tumors were harvested and weighed. Animal experiments were approved by the animal ethics committee of Clinical Medical College, Yangzhou University.

### Statistical analyses

The GraphPad Prism and SPSS software were used to analyze data. Quantitative variables were compared using the Student’s t-test. Relationships between 2 categorical variables were assessed using the Pearsons’ correlation coefficient. Log-rank test and Kaplan-Meier analysis were used to assess the survival rate of patients. Univariate or multivariate hazards were determined with Cox proportional hazards model. *p* ≤ 0.05 was considered to be statistically significant.

## Results

### Expression of GIT1 is elevated in HCC tissues

**Figure 1.**
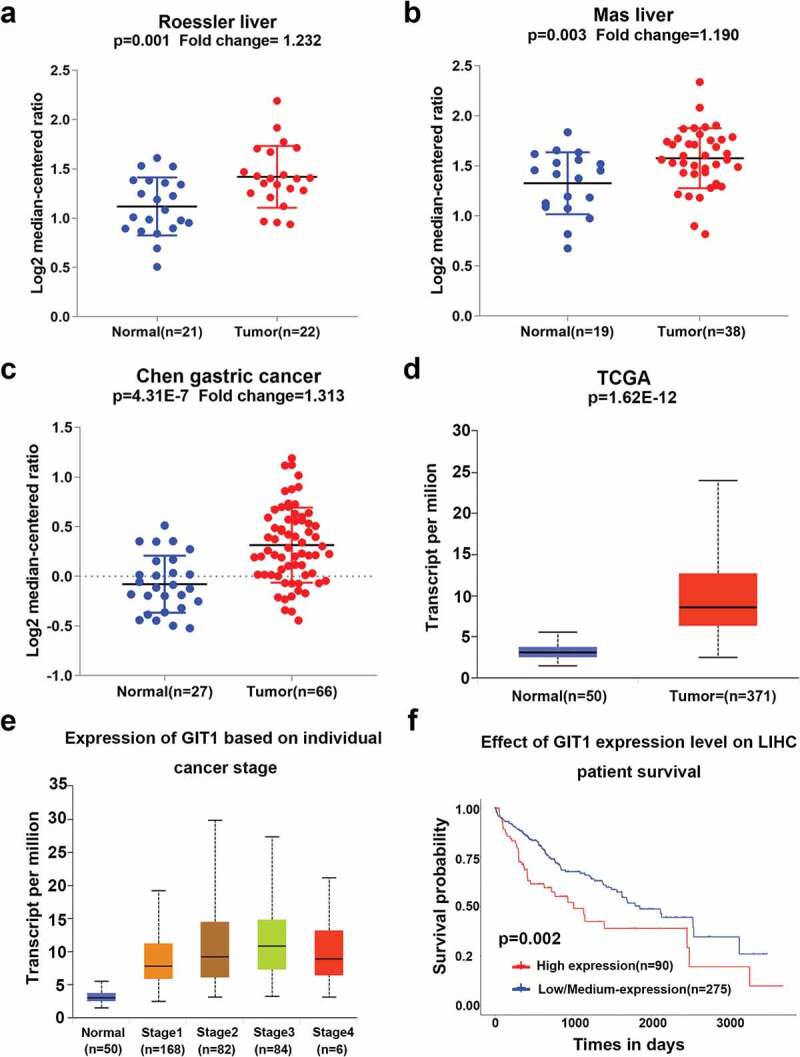
GIT1 expression in human cancers

The mRNA expression levels of GIT1 from the Oncomine database were significantly elevated in tumor tissues when compared to the normal tissues (Figure 1(a,b)). Moreover, GIT1 expression was also found to be elevated in gastric cancer (Figure 1(c)). In 371 HCC tissues and 50 normal liver tissues from TCGA, the expression of GIT1 was consistent with the trend in Oncomine (*p* < 0.001; Figure 1(d)). In addition, the difference in GIT1 expression levels between the normal control group and different clinical stages was also found to be significant (Figure 1(e)). Analysis of TCGA survival data showed that high GIT1 levels were correlated with poor prognosis (*p* = 0.002; Figure 1(f)).

(a–c) The mRNA level of GIT1 in gastric and liver cancers exceeded that of normal tissues based on oncomine database. According to the TCGA database, (d) the expression of GIT1 was different between HCC and normal liver tissues, and (e) was correlated significantly with the clinical stage. (f) Overall survival (OS) curve of HCC patients with high and low expression of GIT1.

**Figure 2.**
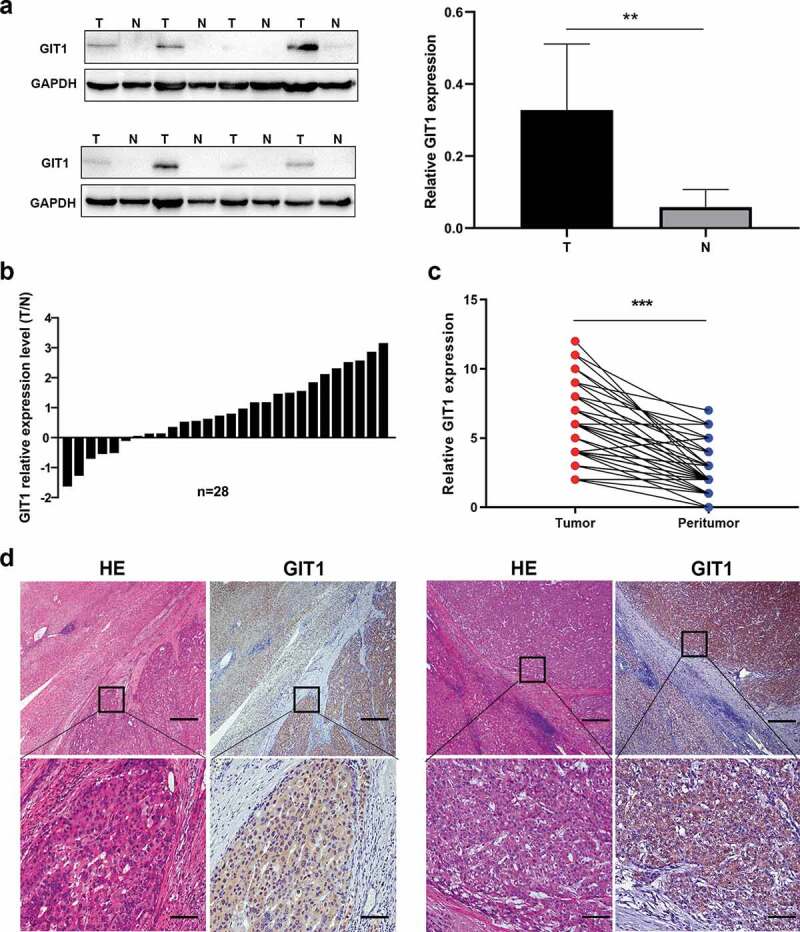
Expression pattern of GIT1 in HCC tissues

Evaluation of GIT1 protein levels in 8 pairs of HCC tumor tissues and adjacent tissues using western blot showed that GIT1 levels were significantly high in HCC tissues (*p* = 0.003; Figure 2(a)). Among 28 pairs HCC tissue and adjacent non-cancer tissues, the RNA expression level of GIT1 was found to be up-regulated in 22 pairs (78.6%) (Figure 2(b)). Similarly, IHC analysis showed that GIT1 staining intensity was markedly higher in HCC tumor tissues (*p* < 0.001; Figure 2(c,d)). These data show that GIT1 is significantly overexpressed in HCC tissues.

(a) GIT1 protein expression in randomly selected HCC tissues and matched adjacent tissues. GAPDH was used as the house-keeping gene. (b) The mRNA expression level of GIT1 in 28 pairs of HCC tissues and their matched peritumor tissues. GIT1 expression was normalized to that of GAPDH. (c) Immunohistochemical staining for GIT1 expression in randomly selected 54 pairs of HCC tissues from the TMA. (d) Representative images of HCC tissues and adjacent matched normal tissues (magnification: 40 ×, 200 ×). Scale bar: 50, 250 μm; **p < 0.01; ***p < 0.001.

### GIT1 expression correlates with HCC cell malignant features

**Figure 3.**
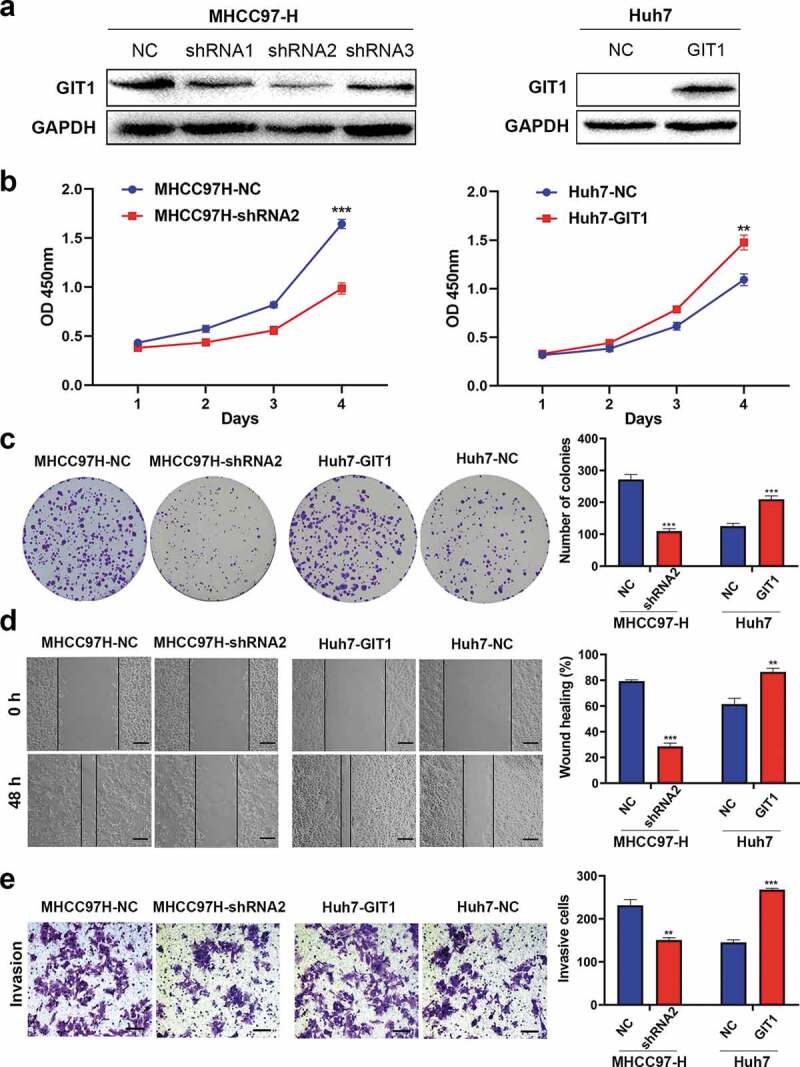
GIT1 overexpression promoted HCC cell invasion, proliferation, and migration

To evaluate the impact of GIT1 on HCC progression, we detected GIT1 expression levels in different HCC cell lines. MHCC97-H was found to have high GIT1 expression levels while Huh7 exhibited low GIT1 expression levels (Supplementary Figure 1). GIT1 expression in MHCC97-H cells was knocked-down by transfecting GIT1-shRNA lentiviral vector. Meanwhile, GIT1 was overexpressed in Huh7 cells with GIT1 cDNA lentiviral vector. Western blotting was used to verify the transfection efficiency (Figure 3(a)). The shRNA with the highest silencing efficiency was chosen for subsequent functional experiments. CCK-8 analysis showed that GIT1 overexpression significantly enhanced cell proliferation at day 4 (Figure 3(b)). The clonogenic assay showed that colony formation in Huh7 cells was significantly improved after GIT1 overexpression (Figure 3(c)). Knockdown of GIT1 in MHCC97-H cells suppressed proliferation and colony formation when compared to the negative control group (Figure 3(b,c)). Furthermore, wound healing and Transwell invasion assays were performed to determine the role of GIT1 in HCC invasion and metastasis. Similar to the previous results, GIT1 upregulation accelerated HCC cell migration and invasion (Figure 3(d,e)).

(a) Successful transfection of lentiviral vector into Huh7 and MHCC97-H cell lines as determined by western blot. GAPDH was used as an internal control. (b) CCK-8 and (c) colony-forming assays were used to assess the ability of cell proliferation. (d) Determination of the migratory ability of HCC cells by wound-healing assay. Scale bar = 100 μm. (e) Measurement of cell invasion ability by Transwell assay. Scale bar = 100 μm. **p < 0.01; ***p < 0.001.

### GIT1 promotes HCC tumorigenesis in vivo

**Figure 4.**
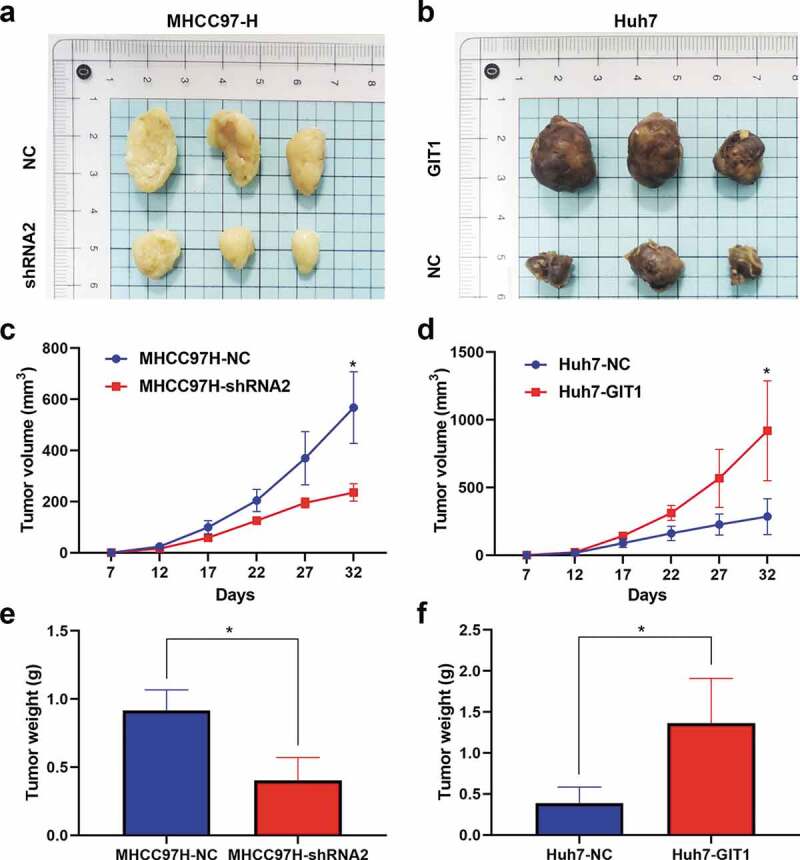
GIT1 promoted HCC tumorigenesis in vivo

We subcutaneously injected Huh7-GIT1, MHCC97H-shRNA2, and corresponding control transfected cells into the flanks of male nude mice to assess the impact of GIT1 on cell proliferation *in vivo*. Tumors were harvested after 32 days for analysis. GIT1 silencing markedly suppressed tumor growth, while its overexpression promoted tumorigenesis in the xenograft model (Figure 4(a,b)). MHCC97H-shRNA2 tumors were light in weight and exhibited a slow growth rate relative to those generated from the negative control cells, while Huh7-GIT1 tumors exhibited larger tumor volumes and greater weights (Figure 4(c–f)). These findings imply that GIT1 has an oncogenic role in HCC development *in vivo*.

(a, b) Representative pictures of tumors derived from nude mice implanted with the MHCC97H-NC, MHCC97H-shRNA2, Huh7-NC, and Huh7-GIT1 cell lines (n = 3). Tumor volume (c, d) and weight (e-f) revealed that GIT1 significantly promoted tumor growth. *p < 0.05.

### GIT1 induces epithelial-mesenchymal transition (EMT) in HCC cells by modulating ERK1/2

**Figure 5.**
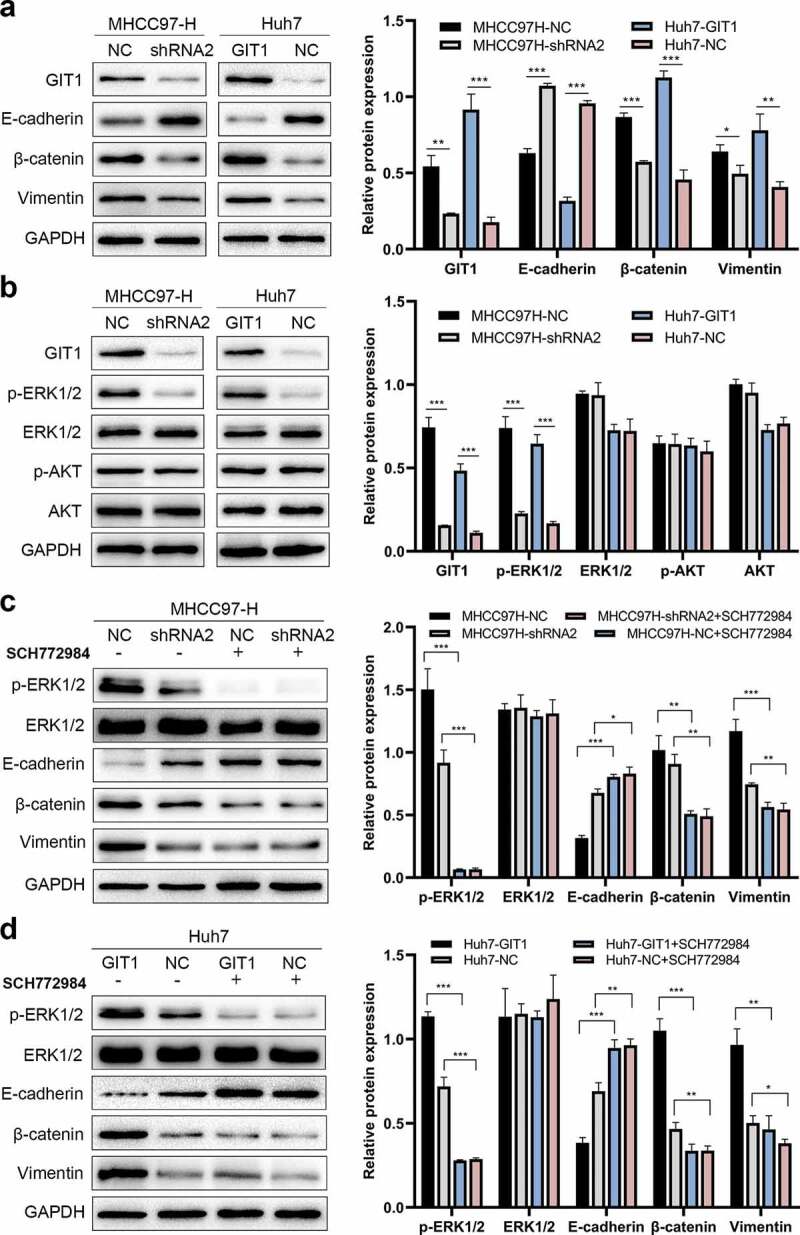
GIT1 promoted EMT in HCC cells through the ERK1/2

Multiple studies have reported that EMT is involved in cancer metastasis. Therefore, we evaluated whether GIT1 enhances HCC invasion and migration through the EMT pathway. Western blot analysis revealed that upon GIT1 overexpression, E-cadherin was suppressed, while β-cadherin and Vimentin were elevated relative to the negative control. Conversely, GIT1 knockdown led to elevated E-cadherin levels while β-cadherin and Vimentin levels were inhibited (Figure 5(a)).

(a) Expression of Vimentin, β-catenin, and E-cadherin, in HCC cells with different GIT1 expression was determined using western blotting assays. (b) Protein levels of p-ERK1/2, ERK1/2, AKT, and p-AKT were analyzed by western blot. (c-d) SCH772984, the inhibitor of ERK1/2, inhibited ERK1/2 signaling and reversed the GIT1-induced EMT.Abbreviations: ERK1/2, extracellular regulated kinase 1/2; p-, phosphorylated.

Next, we sought to identify the signaling pathway regulated by GIT1 and found that p-ERK1/2 levels were suppressed after GIT1 knock-down in MHCC97-H. However, p-AKT levels were not altered. On the contrary, p-ERK1/2 protein levels were enhanced by GIT1 overexpression in Huh7 cells, indicating that GIT1 might promote HCC progression by activating ERK1/2 signaling (Figure 5(b)). To validate these findings, we suppressed ERK1/2 signaling by using a pathway inhibitor (SCH77284). There was a marked reduction in p-ERK1/2 levels in MHCC97H-NC cells, leading to elevated E-cadherin levels and suppressed β-catenin and Vimentin levels (Figure 5(c)). The effects of GIT1 overexpression in Huh7-GIT1 cells treated with SCH77284 on E-cadherin, β-catenin, and Vimentin were similar to those of the control cells treated with SCH77284 (Figure 5(d)). The above results imply that GIT1 promotes tumor progression by inducing EMT through ERK1/2 signaling regulation in HCC.

### High GIT1 levels are associated with poor HCC prognosis

**Figure 6.**
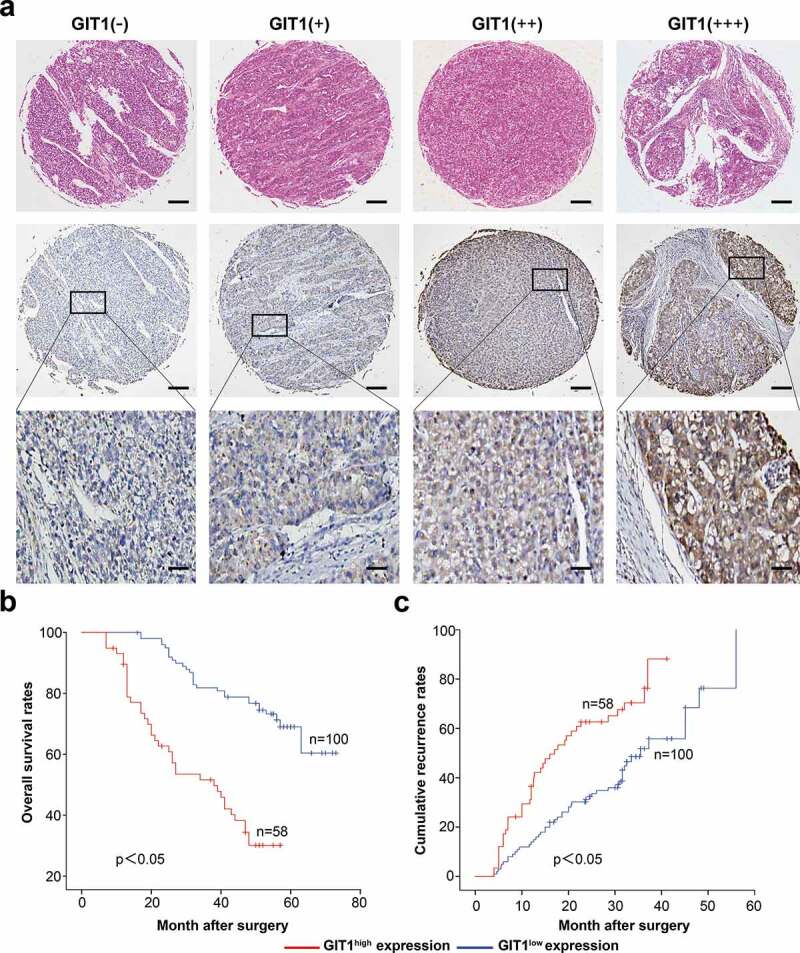
The expression of GIT1 and its prognosis value in 158 HCC patients

GIT1 expression varied significantly in HCC patients from the TMAs (Figure 6(a)). Based on the expression level, they were divided into 2 groups; GIT1^low^ (absent and weak staining) and GIT1^high^ (moderate and strong staining). In further analyses, we found that elevated GIT1 levels correlated with poor prognosis (Figure 6(b,c)). OS rates of HCC patients with GIT1^low^ were significantly higher than patients with GIT1^high^ (*p* < 0.05). The cumulative recurrence rates of HCC patients with GIT1^low^ were markedly lower than GIT1^high^ (*p* < 0.05).

(a) Representative graphs of immunohistochemical and H&E staining for GIT1 in HCC samples: (+++) strong; (++) moderate; (+) low; (-) absence; Scale bar: 50, 250 μm. (b–c) Prognostic significance of GIT1 expression in patients with HCC are illustrated.Abbreviations: H&E, hematoxylin and eosin; OS, overall survival.

The relationship between HCC clinicopathologic features and GIT1 expression was assessed. GIT1 expression was significantly correlated with gender (*p* = 0.019), tumor size (*p* = 0.023), and embolus (*p* = 0.002) (Table 1). However, the poor representation of women in the samples may have contributed to the gender differences. Univariate analysis showed that tumor size (*p* < 0.001), numbers (*p* = 0.042), embolus (*p* = 0.021), and GIT1 expression (*p* < 0.001) were predictors of OS. Tumor size (*p* = 0.003), capsulation (*p* = 0.011), and GIT1 expression (*p* < 0.001) were predictors of cumulative recurrence (Table 2). Multivariate Cox model analysis revealed that GIT1 was an independent prognostic factor for cumulative recurrence (*p* = 0.009) and OS in HCC (*p* < 0.001; Table 2).

**Table 1.** Correlations between GIT1 with clinicopathologic features in 158 HCC patients

Variable		Number of patients		P-value
		GIT1^low^	GIT1^high^	
Gender	Men	79	54	**0.019**
	Women	21	4	
Age, years	≧53	50	25	0.403
	<53	50	33	
HBsAg	Positive	77	48	0.391
	Negative	23	10	
HCV	Positive	14	4	0.176
	Negative	86	54	
Serum TB, μ mol/L	≧17	30	19	0.718
	<17	70	39	
Serum ALB, g/dL	≧3.5	99	55	0.140^a^
	<3.5	1	3	
Serum ALT, U/L	≧75	15	9	0.930
	<75	85	49	
Serum AFP, ng/mL	≧20	60	40	0.260
	<20	40	18	
Cirrhosis	YES	91	51	0.538
	NO	9	7	
Tumor size (diameter, cm)	≧5	35	31	**0.023**
	<5	65	27	
Tumor number	Multiple	15	10	0.710
	Solitary	85	48	
Embolus	YES	15	21	**0.002**
	NO	85	37	
Capsulation	YES	54	27	0.367
	NO	46	31	
Tumor differentiation	ⅠⅠⅠ/Ⅳ	31	12	0.160
	Ⅰ/Ⅱ	69	46	

**Notes**: a, Fisher’s exact test. GIT1^high^≥50% staining; GIT1^low^<50% staining. Statistically significant values are shown in bold. **Abbreviations**: HBsAg, hepatitis B surface antigen; HCV, hepatitis C virus; AFP, α-fetoprotein; TB, total bilirubin; ALB, albumin; ALT, alanine aminotransferase;

**Table 2.** Univariate and multivariate analyses of factors associated with recurrence and survival

	Overall survival			Cumulative recurrence		
Variable	Univariate	Multivariate		Univariate	Multivariate	
	P-value	HR (95% CI)	P-value	P-value	HR (95% CI)	P-value
Gender (men vs women)	0.140		NA	0.059		NA
Age, years (≧53 vs, <53)	0.500		NA	0.498		NA
HBsAg (positive vs negative)	0.770		NA	0.613		NA
HCV (positive vs negative)	0.978		NA	0.852		NA
AFP, ng/ml (≧20 vs, <20)	0.170		NA	0.133		NA
Serum TB, μmol/L (≧17 vs, <17)	0.920		NA	0.983		NA
Serum ALB, g/dL (≧3.5 vs, <3.5)	0.693		NA	0.224		NA
Serum ALT, U/L (≧75 vs, <75)	0.347		NA	0.467		NA
Cirrhosis (yes vs no)	0.388		NA	0.275		NA
Tumor size (diameter, cm) (≧5 vs, <5)	<0.001	2.454(1.393-4.322)	0.002	0.003	2.013(1.237-3.276)	0.005
Tumor number (multiple vs solitary)	0.042		NS	0.066		NA
Embolus (yes vs no)	0.021		NS	0.118		NA
Capsulation (yes vs no)	0.362		NA	0.011	1.790(1.127-2.844)	0.014
Tumor differentiation (ⅠⅠⅠ/Ⅳ vs, Ⅰ/Ⅱ)	0.700		NA	0.831		NA
GIT1 density (<50% vs, ≧50%)	<0.001	3.794(2.119-6.794)	<0.001	<0.001	1.943 (1.185-3.188)	0.009

**Abbreviations**: HBsAg, hepatitis B surface antigen; HCV, hepatitis C virus; AFP, α-fetoprotein; TB, total bilirubin; ALB, albumin; ALT, alanine aminotransferase; NS, not significant; NA, not adopt.

## Discussion

To improve the prognosis of HCC patients, effective early detection, diagnosis, and treatment strategies are needed. Aiming to identify novel therapeutic targets, studies have been aimed at elucidating the molecular basis of HCC progression. In this study, GIT1 expression in HCC tissues exceeded that in adjacent non-tumor tissues. It was shown to accelerate metastasis, proliferation, HCC cell invation, and promoted tumorigenesis in vivo. Moreover, our findings indicated that GIT1 enhances tumor progression by modulating EMT and is a predictor for poor prognosis in HCC. Moreover, we found that GIT1 might positively regulate EMT by activating ERK1/2 signaling. and further confirmed the relevance of GIT1 with malignant phenotypes and prognosis in clinical data. All these findings highlighted GIT1 as a potential novel therapeutic target and an independent prognostic factor in HCC.

GIT1 interacts with various signaling molecules and regulates many biological processes, such as cell mobility. GIT1 regulates Paxillin and PIX assembly in focal adhesions to influence neurons, osteoblasts and vascular smooth muscle cell migration [12,26–29]. In addition, GIT1 also plays an important role in other malignant tumors. For instance, it promotes lung cancer cell invasion and migration by modulating Rac1/Cdc42 activity [23]. In osteosarcoma, GIT1 silencing effectively suppresses tumor growth, invasion, and angiogenesis [30]. GIT1 is targeted by miR-149-5p to suppress the invasion and proliferation of medullary thyroid carcinoma cells [31]. Besides, in gastric cancer, Methyl-CpG binding protein 2 (MeCP2) facilitates cell proliferation and cell-cycle progression by activating MEK1/2-ERK1/2 signaling through GIT1 upregulation [25]. Here, we clarify the role of GIT1 in HCC cells and tissues, especially emphasize its clinical significance for HCC patients. GIT1 was highly expressed in HCC and enhanced its progression. GIT1 silencing inhibited the invasion, migration, and tumorigenic potential of HCC cells. Clinical significance analysis showed that GIT1 was associated with tumor size and embolus, and HCC patients with GIT1^low^ expression exhibited longer survival outcomes than those with GIT1^high^. These results implied that GIT1 might function as a potential prognostic biomarker and therapeutic target for HCC.

Accumulating evidence has revealed that EMT plays a critical role during the metastasis of a variety of tumors, including HCC. During EMT, epithelial cells differentiate to acquire mesenchymal features, such as migration and invasion [32]. In this process, tumor cells dissociate from their primary site and as free cells migrate and invade distant sites. EMT induction is characterized by elevated β-catenin and Vimentin levels as well as low E-cadherin levels. GIT1 is involved in the regulation of tumor cell invasion and migration by modulating EMT [24,33]. In our findings, E-cadherin, β-catenin, and Vimentin expression levels were affected by GIT1. Overexpression of GIT1 enhanced the expression of β-catenin and Vimentin but inhibited the expression of E-cadherin, indicating that GIT1 may mediate HCC metastasis through EMT.

GIT1, as a scaffold protein, is co-localized with ERK1/2 and activates ERK1/2 signaling [18,34]. The ERK1/2 pathway is involved in cell invasion and migration, and is activated in HCC [35]. Therefore, we determined whether GIT1 promotes EMT through the ERK1/2 signaling pathway. We found that ERK1/2, but not AKT, was phosphorylated upon GIT1 overexpression in HCC cells. Moreover, GIT1 was shown to promote EMT in HCC cells by activating ERK1/2 signaling, but the underlying mechanisms by which GIT1 affect ERK1/2 activation has not been established. Whether GIT1 acts as a scaffold and directly interacts with ERK1/2 should be studies further. However, SCH772984, an ERK1/2 inhibitor, attenuated the EMT changes induced by GIT1 overexpression, which implied that ERK1/2 signaling activation was vital for GIT1-mediated EMT. Although more mechanistic studies are needed, therapeutic strategies targeting the ERK1/2 pathway might be feasible for inhibiting HCC progression.

## Conclusion

All in all, GIT1 is elevated in HCC where it enhances the proliferation, invasion, and migration of HCC cells by modulating EMT through ERK1/2 signaling. Inhibition of GIT1 suppresses EMT and HCC progression. Therefore, GIT1 maybe a novel therapeutic target for HCC.

## Supplementary Material

Supplemental Material

## Highlights

(1) GIT1 expression is elevated in HCC tissues than that in adjacent non-tumor tissues.

(2) GIT1 accelerated the proliferation, migration and invasion of HCC cells.

(3) GIT1 promotes EMT in HCC cells by modulating ERK1/2.

(4) GIT1 expression is an independent prognostic factor for cumulative HCC recurrence and OS.

## Abbreviations

HCC, hepatocellular carcinoma; GIT1, G-protein-coupled receptor (GPCR)-kinase interacting protein-1; EMT, epithelial-mesenchymal transition; TMA, tissue microarray; OS, overall survival; ERK1/2, extracellular regulated kinase 1/2; p-ERK1/2, phosphorylation ERK1/2; ARFGAP, ADP ribosylation factor GTPase-activating protein;

## References

[1] Siegel RL, Miller KD, Jemal A. Cancer statistics, 2019. CA Cancer J Clin. 2019;69:7–34. 30620402 10.3322/caac.21551

[2] Villanueva A. Hepatocellular carcinoma. N Engl J Med. 2019;380:1450–1462. 30970190 10.1056/NEJMra1713263

[3] Lurje I, Czigany Z, Bednarsch J, et al. Treatment strategies for hepatocellular carcinoma (-) a multidisciplinary approach. Int J Mol Sci. 2019;20. DOI:10.3390/ijms20061465 PMC6470895 30909504

[4] Zhong JH, Ma L, Li LQ. Postoperative therapy options for hepatocellular carcinoma. Scand J Gastroenterol. 2014;49:649–661. 24716523 10.3109/00365521.2014.905626

[5] Mazzola A, Costantino A, Petta S, et al. Recurrence of hepatocellular carcinoma after liver transplantation: an update. Futur Oncol. 2015;11:2923–2936. 10.2217/fon.15.239 26414336

[6] Hoefen RJ, Berk BC. The multifunctional GIT family of proteins. J Cell Sci. 2006;119:1469–1475. 16598076 10.1242/jcs.02925

[7] Premont RT, Claing A, Vitale N, et al. beta2-Adrenergic receptor regulation by GIT1, a G protein-coupled receptor kinase-associated ADP ribosylation factor GTPase-activating protein. Proc Natl Acad Sci U S A. 1998;95:14082–14087. 9826657 10.1073/pnas.95.24.14082 PMC24330

[8] Martyn AC, Toth K, Schmalzigaug R, et al. GIT1 regulates synaptic structural plasticity underlying learning. PLoS One. 2018;13:e0194350. 29554125 10.1371/journal.pone.0194350 PMC5858814

[9] Li YS, Qin LX, Liu J, et al. GIT1 enhances neurite outgrowth by stimulating microtubule assembly. Neural Regen Res. 2016;11:427–434. 27127481 10.4103/1673-5374.179054 PMC4829007

[10] Fiuza M, Gonzalez-Gonzalez I, Perez-Otano I. GluN3A expression restricts spine maturation via inhibition of GIT1/Rac1 signaling. Proc Natl Acad Sci U S A. 2013;110:20807–20812. 24297929 10.1073/pnas.1312211110 PMC3870762

[11] Won H, Mah W, Kim E, et al. GIT1 is associated with ADHD in humans and ADHD-like behaviors in mice. Nat Med. 2011;17:566–572. 21499268 10.1038/nm.2330

[12] Pang J, Xu X, Wang X, et al. G-protein-coupled receptor kinase interacting protein-1 mediates intima formation by regulating vascular smooth muscle proliferation, apoptosis, and migration. Arter Thromb Vasc Biol. 2013;33:999–1005. 10.1161/ATVBAHA.112.300966 PMC3703521 23430614

[13] Pang J, Hoefen R, Pryhuber GS, et al. G-protein-coupled receptor kinase interacting protein-1 is required for pulmonary vascular development. Circulation. 2009;119:1524–1532. 19273721 10.1161/CIRCULATIONAHA.108.823997 PMC2732662

[14] Chen P, Gu WL, Gong MZ, et al. GIT1 gene deletion delays chondrocyte differentiation and healing of tibial plateau fracture through suppressing proliferation and apoptosis of chondrocyte. BMC Musculoskelet Disord. 2017;18:320. 28754105 10.1186/s12891-017-1653-7 PMC5534123

[15] Manabe R, Kovalenko M, Webb DJ, et al. GIT1 functions in a motile, multi-molecular signaling complex that regulates protrusive activity and cell migration. J Cell Sci. 2002;115:1497–1510. 11896197 10.1242/jcs.115.7.1497

[16] Penela P, Nogues L, Mayor F Jr. Role of G protein-coupled receptor kinases in cell migration. Curr Opin Cell Biol. 2014;27:10–17. 24680425 10.1016/j.ceb.2013.10.005

[17] Wilson E, Leszczynska K, Poulter NS, et al. RhoJ interacts with the GIT-PIX complex and regulates focal adhesion disassembly. J Cell Sci. 2014;127:3039–3051. 24928894 10.1242/jcs.140434 PMC4106786

[18] Zhang N, Cai W, Yin G, et al. GIT1 is a novel MEK1-ERK1/2 scaffold that localizes to focal adhesions. Cell Biol Int. 2009;34:41–47. 19947948 10.1042/CBI20090016 PMC3125965

[19] Li L, Tang P, Zhou Z, et al. GIT1 regulates angiogenic factor secretion in bone marrow mesenchymal stem cells via NF-kappaB/Notch signalling to promote angiogenesis. Cell Prolif. 2019;52:e12689. 31502302 10.1111/cpr.12689 PMC6869488

[20] Zhang LQ, Zhao GZ, Xu XY, et al. Integrin-beta1 regulates chondrocyte proliferation and apoptosis through the upregulation of GIT1 expression. Int J Mol Med. 2015;35:1074–1080. 25715677 10.3892/ijmm.2015.2114

[21] Zhao SJ, Kong FQ, Cai W, et al. GIT1 contributes to autophagy in osteoclast through disruption of the binding of Beclin1 and Bcl2 under starvation condition. Cell Death Dis. 2018;9:1195. 30546041 10.1038/s41419-018-1256-8 PMC6294144

[22] Huang WC, Chan SH, Jang TH, et al. miRNA-491-5p and GIT1 serve as modulators and biomarkers for oral squamous cell carcinoma invasion and metastasis. Cancer Res. 2014;74:751–764. 24335959 10.1158/0008-5472.CAN-13-1297

[23] Chang JS, Su CY, Yu WH, et al. GIT1 promotes lung cancer cell metastasis through modulating Rac1/Cdc42 activity and is associated with poor prognosis. Oncotarget. 2015;6:36278–36291. 26462147 10.18632/oncotarget.5531 PMC4742177

[24] Dong Y, Chang C, Liu J, et al. Targeting of GIT1 by miR-149* in breast cancer suppresses cell proliferation and metastasis in vitro and tumor growth in vivo. Onco Targets Ther. 2017;10:5873–5882. 29270025 10.2147/OTT.S144280 PMC5729835

[25] Zhao LY, Tong DD, Xue M, et al. MeCP2, a target of miR-638, facilitates gastric cancer cell proliferation through activation of the MEK1/2-ERK1/2 signaling pathway by upregulating GIT1. Oncogenesis. 2017;6:e368. 28759023 10.1038/oncsis.2017.60 PMC5541712

[26] Li Y, Wu X, Liu Z, et al. Integrin-mediated signaling via Paxillin-GIT1-PIX promotes localized Rac activation at the leading edge and cell migration. J Cancer. 2020;11:345–352. 31897230 10.7150/jca.32853 PMC6930425

[27] Za L, Albertinazzi C, Paris S, et al. betaPIX controls cell motility and neurite extension by regulating the distribution of GIT1. J Cell Sci. 2006;119:2654–2666. 16787945 10.1242/jcs.02996

[28] Zhang N, Hu Z, Yin G. [Mechanism of G protein coupled receptor kinase interacting protein 1 RNA hairpin inhibiting osteoblasts migration]. Zhongguo Xiu Fu Chong Jian Wai Ke Za Zhi. 2007;21:1–5. 17304992

[29] Majumder S, Sowden MP, Gerber SA, et al. G-protein-coupled receptor-2-interacting protein-1 is required for endothelial cell directional migration and tumor angiogenesis via cortactin-dependent lamellipodia formation. Arter Thromb Vasc Biol. 2014;34:419–426. 10.1161/ATVBAHA.113.302689 PMC4295836 24265417

[30] Zhang Z, Hu P, Xiong J, et al. GIT1 reduces the growth, invasion, and angiogenesis of osteosarcoma. Cancer Manag Res. 2018;10:6445–6455. 30555255 10.2147/CMAR.S181066 PMC6278701

[31] Ye X, Chen X. miR-149-5p inhibits cell proliferation and invasion through targeting GIT1 in medullary thyroid carcinoma. Oncol Lett. 2019;17:372–378. 30655777 10.3892/ol.2018.9628 PMC6313157

[32] Thiery JP, Acloque H, Huang RY, et al. Epithelial-mesenchymal transitions in development and disease. Cell. 2009;139:871–890. 19945376 10.1016/j.cell.2009.11.007

[33] Li J, Wang Q, Wen R, et al. MiR-138 inhibits cell proliferation and reverses epithelial-mesenchymal transition in non-small cell lung cancer cells by targeting GIT1 and SEMA4C. J Cell Mol Med. 2015;19:2793–2805. 26283050 10.1111/jcmm.12666 PMC4687704

[34] Yin G, Zheng Q, Yan C, et al. GIT1 is a scaffold for ERK1/2 activation in focal adhesions. J Biol Chem. 2005;280:27705–27712. 15923189 10.1074/jbc.M502271200

[35] Schmitz KJ, Wohlschlaeger J, Lang H, et al. Activation of the ERK and AKT signalling pathway predicts poor prognosis in hepatocellular carcinoma and ERK activation in cancer tissue is associated with hepatitis C virus infection. J Hepatol. 2008;48:83–90. 17998146 10.1016/j.jhep.2007.08.018

