# Supplementary material for: GIT1 overexpression promotes epithelial-mesenchymal transition and predicts poor prognosis in hepatocellular carcinoma
Source: Bioengineered. 2020 Dec 21;12(1):30–43. doi: 10.1080/21655979.2020.1855914 (PMC8806235; doi:10.1080/21655979.2020.1855914)
Supplement: Supplemental Material [file KBIE_A_1855914_SM5672.docx]

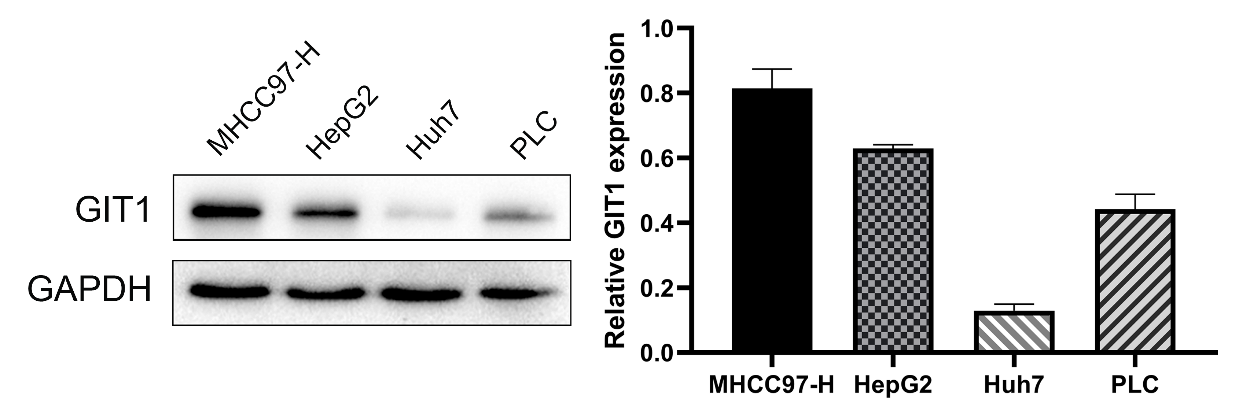


Supplementary Figure 1: GIT1 protein levels in 4 HCC cell lines were measured by western blot (MHCC97-H, HepG2, Huh7, PLC).
